# EXAMINING THE DETERMINANTS AND OUTCOMES OF SUBJECTIVE AGING

**DOI:** 10.1093/geroni/igz038.1411

**Published:** 2019-11-08

**Authors:** Allyson F Brothers, Serena Sabatini, Shevaun D Neupert

**Affiliations:** 1 Colorado State University, Fort Collins, Colorado, United States; 2 Centre for Research in Ageing and Cognitive Health, University of Exeter, Exeter, United Kingdom, University of Exeter, United Kingdom; 3 North Carolina State University, Raleigh, North Carolina, United States

## Abstract

Given a growing body of evidence for the developmental relevance of the perceived experience of aging and for the presence of interindividual variability in the way people experience aging, this symposium examines the determinants and outcomes of various subjective aging constructs. This session will explore the role of various psychological variables in explaining variability in subjective aging experiences. Consequences of various subjective aging concepts on cognitive functioning, emotional and physical well-being will also be discussed. The first two presentations examine Attitudes Toward Own Aging (ATOA). Kornadt, Siebert and Wahl will address the developmental co-dynamics of personality and ATOA across the second half of life. Siebert and Wahl will examine the associations of ATOA with subjective and objective cognitive functioning. The last two presentations focus on awareness of age-related change (AARC). Sabatini, Silarova, Collins, Martyr, Ballard, Anstey, Kim & Clare will present findings from a systematic-review and meta-analysis synthesizing and quantifying associations of awareness of age-related change (AARC) with emotional and physical well-being and cognitive functioning. Finally, Rothermund and de Paula Couto will show how both the experience of positive and/or negative changes (gains and losses) and the presence of positive and/or negative age stereotypes predict individual’s attributions of change to age. This last presentation will also examine how together the presence of change and attribution of change to age predict developmental outcomes. The symposium will conclude with summarizing remarks from the discussant who suggests possible directions for future research on determinants and outcomes of perceived experience of aging.

